# Glycosystems in nanotechnology: Gold glyconanoparticles as carrier for anti-HIV prodrugs

**DOI:** 10.3762/bjoc.10.136

**Published:** 2014-06-12

**Authors:** Fabrizio Chiodo, Marco Marradi, Javier Calvo, Eloisa Yuste, Soledad Penadés

**Affiliations:** 1 Laboratory of GlycoNanotechnology, Biofunctional Nanomaterials Unit, CIC biomaGUNE, Paseo Miramón 182, 20009, San Sebastián, Spain; 2 Networking Research Center on Bioengineering, Biomaterials and Nanomedicine (CIBER-BBN), Paseo Miramón 182, 20009, San Sebastián, Spain; 3 Technological Platform of Mass Spectrometry, CIC biomaGUNE, Paseo Miramón 182, 20009, San Sebastián, Spain; 4 AIDS Research Unit, Institut d'Investigacions Biomediques August Pi i Sunyer, Barcelona, Spain; 5 HIVACAT, Barcelona, Spain

**Keywords:** drug-delivery system, gold glyconanoparticles, HAART, HIV, multivalent glycosystems, reverse transcriptase inhibitors

## Abstract

The therapeutic approach for the treatment of HIV infection is based on the highly active antiretroviral therapy (HAART), a cocktail of antiretroviral drugs. Notwithstanding HAART has shown different drawbacks like toxic side effects and the emergence of viral multidrug resistance. Nanotechnology offers new tools to improve HIV drug treatment and prevention. In this scenario, gold nanoparticles are an interesting chemical tool to design and prepare smart and efficient drug-delivery systems. Here we describe the preparation and antiviral activity of carbohydrate-coated gold nanoparticles loaded with anti-HIV prodrug candidates. The nucleoside reverse transcriptase inhibitors abacavir and lamivudine have been converted to the corresponding thiol-ending ester derivatives and then conjugated to ~3 nm glucose-coated gold nanoparticles by means of “thiol-for-thiol” ligand place exchange reactions. The drugs-containing glyconanoparticles were characterized and the pH-mediated release of the drug from the nanoparticle has been determined. The antiviral activity was tested by evaluating the replication of NL4-3 HIV in TZM-bl infected cells. The proof-of-principle presented in this work aims to introduce gold glyconanoparticles as a new multifunctional drug-delivery system in the therapy against HIV.

## Introduction

Acquired immune deficiency syndrome (AIDS), caused by human immunodeficiency virus type-1 (HIV-1) [1] continues to be a major leading pandemic disease worldwide with approximately 34 million people living with HIV [2]. Due to its incredible genetic variance and the specificity for CD4+ T cells, this virus is responsible for 800.000 deaths per year. In addition to sexual preventions, the strategies used to inhibit viral replication in human CD4+ T cells consist in the highly active antiretroviral therapy (HAART) [3] and the design of a vaccine that should protect people among all the different HIV strains [4–5]. Although great results have been obtained by the use of the HAART regimes since 1996, there are still several problems to solve, such as toxic side-effects of the HAART drugs and the emergence of multidrug resistance. Nowadays the safest prevention against sexual infection relies on physical barriers, but recently a new type of protection based on microbicides has started to be developed. Microbicides are a new class of chemical–physical barrier in clinical development that can be directly applied to the vagina or rectum before sexual intercourses in order to prevent the transmission of HIV [6]. Recently, a conventional anti-HIV drug used for HAART was explored as potential microbicide. A gel formulation containing 1% of the reverse transcriptase inhibitor tenofovir has shown good results in the prevention of HIV infections of women in South Africa [7].

One of the greatest challenges of antiretroviral and microbicide therapy is to develop drug-delivery systems (DDSs) with high efficacy and therapeutic selectivity [8] to overcome the drawbacks of HAART. Nanotechnology allows the construction of novel systems that could bring changes in this scenario. Over the last years, different nano-constructions have been designed as prophylactic agents against HIV. Some of these nanomaterials like polymeric nanoparticles, lipid nanoparticles and nanofibers have shown the ability to improve solubility, stability and permeability of anti-HIV drugs [9–10], but also to reduce the viral load by the activation of latently infected CD4+ T-cells [11].

Gold nanoparticles have been explored in biomedicine as multivalent and multifunctional scaffolds [12–13]. Thanks to their relative inertness and low toxicity gold nanoparticles have been widely explored to conjugate biomolecules on their surface, because the chemistry of their surface is easy to control [12]. The application of gold nanoparticles as a DDS is an expanding field due to the inert properties of the gold core, their controlled fabrication, and multifunctionality [14]. This last property allows the design of particles simultaneously containing multiple chemotherapeutics and targeting moieties. Few studies have described the application of gold nanoparticles for HIV treatment. In 2008 gold nanoparticles were used as carrier for an anti-HIV drug [15]. An inactive derivative of the inhibitor TAK-779 (the active part of the drug was modified to link it to the gold surface) was multimerized on gold nanoparticles that showed surprisingly anti-HIV activity, probably due to the high-local concentration of the drug derivative on the gold surface. Other inorganic nanomaterials have also been explored as carriers for therapeutic drugs against HIV. For example, silver nanoparticles coated with poly(vinyl)pyrrolidone were found to be effective against different HIV-strains [16]. Aptamer-conjugated gold nanoparticles were also exploited as effective inhibitors of viral enzymes [17].

We have previously described the usefulness of carbohydrate-coated gold nanoparticles (GNPs) as a carrier for different structures related to HIV envelope [18]. GNPs coated with oligomannosides of the gp120 (*manno*-GNPs) were able to inhibit the DC-SIGN-mediated HIV-1 *trans*-infection of human T-cells [19] and gold glyconanoparticles coated with sulfated ligands showed to interfere with the adhesion/fusion of HIV during its entry [20]. Our methodology for preparing GNPs allows the construction of particles simultaneously containing carbohydrates, peptides and targeting molecules in a controled way [21]. The use of biocompatible gold glyconanoparticles as scaffolds for the antiviral drugs could bring some important benefits such as the improvement of the solubility in water and biological media of the drugs and the improvement of cellular uptake due to the presence of carbohydrates on the GNPs. In addition a local increase of the drug concentration on the gold surface could also improve their antiviral activity. We reasoned that the presence of multiple antiretroviral molecules on carbohydrate-coated gold nanoparticles could lead to a drug-delivery system and/or microbicides able to inhibit viral replication or to prevent sexual infection. We have previously demonstrated that glucose-coated gold nanoparticles are water-soluble and non-cytotoxic to different cell lines at the tested concentrations [22]. Glucose-coated nanomaterials have been proposed as good intracellular delivery tool and the internalization and uptake of glucose-coated nanoparticles have been described on different cell lines [23–26]. In addition glucose-coated gold nanoparticles did not elicit any immune response in animal models [27–28]. We thus decided to use them as a scaffold to insert antiretroviral drugs to construct new multivalent anti-HIV systems.

Here we describe the preparation of anti-HIV prodrug candidates and their assembly on ~3 nm glucose-coated gold nanoparticles as a potential drug-delivery system. As antiviral drugs, the nucleoside analog reverse transcriptase inhibitors (NRTIs) abacavir (ABC) and lamivudine (3TC) were selected. NRTIs are drugs that compete in the cytoplasm as triphosphates with endogenous nucleoside substrates acting as chain terminators in the DNA polymerisation reaction catalyzed by HIV-1 RT [3]. Both drugs were transformed in ester derivatives to prepare the GNPs. The pH-mediated release of the drugs from the GNPs surface was evaluated and cellular experiments demonstrated that abacavir and lamivudine ester derivatives tailored onto the gold gluconanoparticles have an antiviral activity similar to the free drugs.

## Results and Discussion

### Preparation of anti-HIV prodrug-GNPs

**Figure 1:**
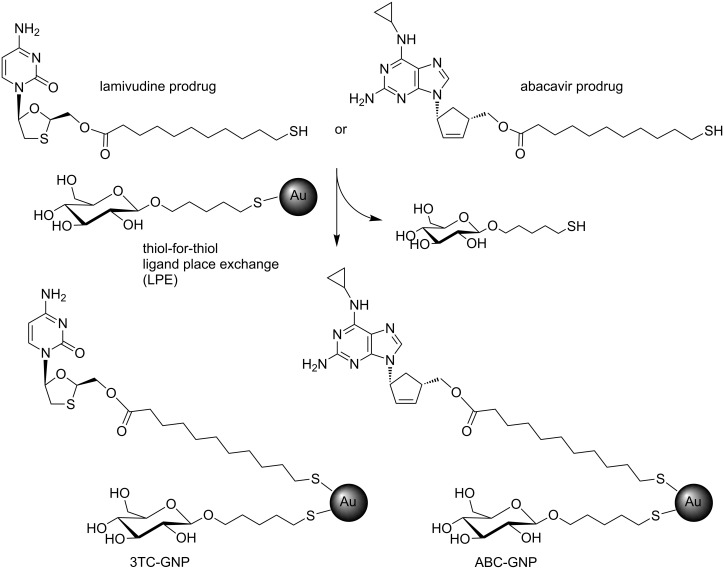
The prepared lamivudine (3TC) and abacavir (ABC) potential prodrugs and the corresponding 3TC- and ABC-GNPs prepared by ligand place exchange (LPE) reactions. Glucose-GNPs were incubated for 22 h with 0.1 equiv of ABC or 3TC thiol-ending drug derivatives. The reaction conditions allowed the “thiol-for-thiol” ligand exchange on the gold surface by replacing some glucose ligands on the glucose-GNPs with the prodrug candidates.

As a proof-of-principle for a further exploration of gold glyconanoparticles as drug-delivery system, we prepared glucose-coated gold nanoparticles and functionalized them with in clinical use antiviral drugs abacavir (ABC) and lamivudine (3TC). The drugs were functionalized at the primary hydroxy groups with 11-mercaptoundecanoic acid to obtain the prodrug candidate with an easy hydrolysable ester group that allows the release of the drug from the GNPs by enzymatic or pH mediated hydrolysis. 11-Mercaptoundecanoic acid was chosen as bifunctional aliphatic linker between the drugs and the gold nanoparticles. Aliphatic ester prodrugs of the anti-HIV drug zidovudine have previously shown to promote intestinal lymph transport (a major reservoir for HIV) [29] and some alkyl and alkyloxyalkyl esters of nucleotides or acyclic nucleoside phosphonates have been explored in clinical studies [30]. In order to obtain the ester derivatives, 11-(acetylthio)undecanoic acid, obtained from 11-bromoundecanoic acid and potassium thioacetate [31], was reacted with ABC and 3TC in DMF in the presence of 1-ethyl-3-(3-dimethylaminopropyl)carbodiimide (EDC) and 4-dimethylaminopyridine (DMAP) to obtain the ester derivative in ~75% yield. After purification, the protecting group of the thiol was removed with hydrazine acetate to give the corresponding ester prodrug candidates with a free thiol-ending group fundamental for their gold chemo-adsorption (Figure 1 and Supporting Information File 1).

Abacavir (ABC) and lamivudine (3TC) were functionalized at the primary hydroxy groups through an ester bond that will be cleaved by cellular esterase activity or acid conditions in the cellular medium (or vaginal acidic pH). The primary hydroxy group of these NRTIs is fundamental for their antiviral activity: its intracellular enzymatic phosphorylation will form triphosphate derivatives that are the real chain terminators of HIV reverse transcriptase [3].

Due to the presence of an ester group in the prepared drug derivatives, NaBH_4_ could not be used as reducing agent for the in situ preparation of these gold nanoparticles [32–33]. The ABC- and 3TC-GNPs were then prepared by the so-called “thiol-for-thiol” ligand place exchange (LPE) reaction [34]. The LPE reaction methodology allows the insertion of thiol ending ligands (the thiol-ending prodrug candidates) on pre-formed GNPs (GNPs fully covered by a glucose conjugate [35]) by a “thiol-for-thiol” exchange on the gold surface (Figure 1) following a reported methodology [24]. Preformed glucose-GNPs were incubated with 0.1 equivalents of ABC or 3TC conjugate with respect to the glucose conjugates on the GNP. This amount allowed the insertion of ~10% of the thiol-ending drugs. After precipitation and washings with EtOH, the GNPs were dissolved in a 90:10 mixture of water/DMSO to ensure a better GNPs water-dispersion that was also used for the cellular experiments. The GNPs dimension was evaluated by electron microscopy (Supporting Information File 1) showing an average gold diameter of ~3 nm. The GNPs contain around 10% of ABC or 3TC were analysed by HPLC and mass spectrometry (see next paragraph). The ester derivatives were not detected in the EtOH washings after the GNPs precipitation (by MALDI–MS and ^1^H NMR) indicating that practically all the drug conjugates were linked on the gold surface.

### Drug quantification and release of the drug from GNPs

**Figure 2:**
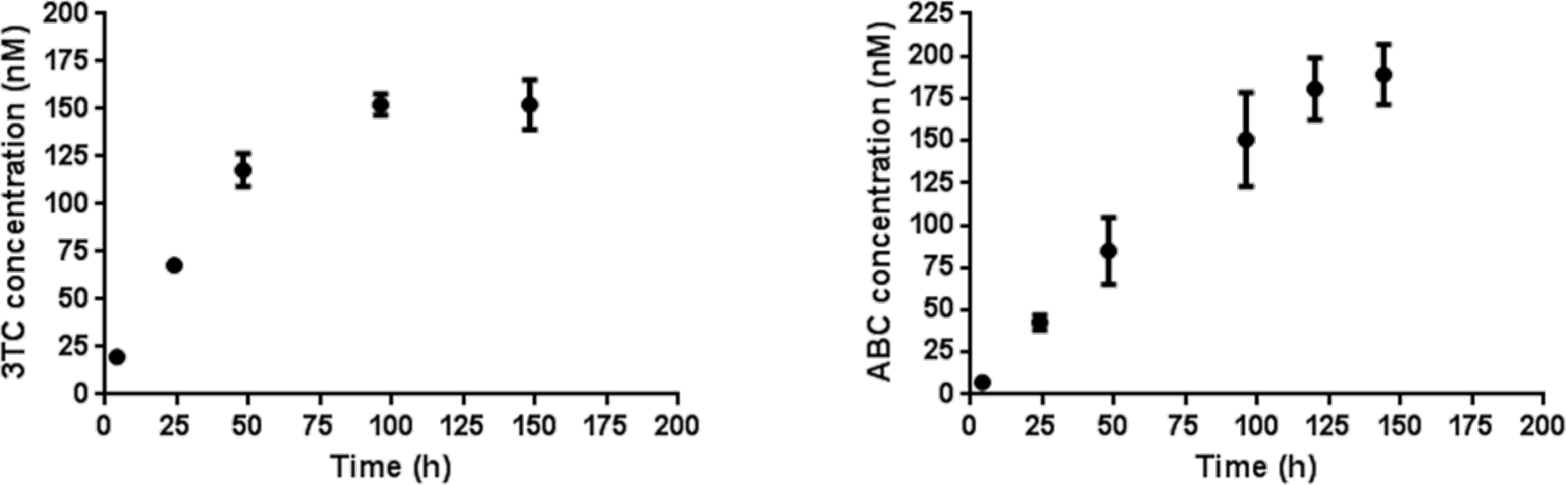
Time course release of free 3TC and ABC from the corresponding GNPs in 1 N HCl, detected by HPLC–MS measurements. Left: Release of 3TC from 2 µg/mL 3TC-GNPs for ~150 h. Right: release of ABC from 2 µg/mL ABC–GNPs for 170 h until a stable drug concentration in the release medium is reached. Both experiments were performed in triplicate.

We studied the stability of the GNPs containing ABC or 3TC (around 10%) in 1 N HCl at different times by liquid chromatography–mass spectrometry (LC–MS, Figure 2). A solution of drugs-GNPs (2 mg/mL) in water was treated with 1 N HCl and 1:1000 dilution aliquots (10 μL) of the GNP solutions were injected into the chromatograph. The free drugs were quantified by mass spectrometry with an internal standard (for detailed ion chromatograms and mass spectra see Supporting Information File 1). In the absence of HCl, the GNPs did not release the drugs showing no peaks in the LC–MS spectra. The pH-mediated delivery of the drugs from the GNPs was followed for 2–3 days until a plateau in the kinetic curve of the drug release was reached (Figure 2). Calibration curves of the free drugs were performed in triplicate by LC–MS (Supporting Information File 1). The release of the drug from a 2 µg/mL GNP dilution after 150–170 h was estimated to be around 150–200 nM from the LC–MS quantification. These experiments were performed in triplicate and repeated with two different GNP batches showing similar results. The pH-mediated release confirmed the estimation of ~10% of the drug on the gold surface and from these results the estimated amount of drug per 1 mg of GNPs was calculated to be ~0.1 μmol (the detailed calculation is given in Supporting Information File 1).

### Cellular experiments with lamivudine (3TC) and abacavir (ABC)-GNPs

**Figure 3:**
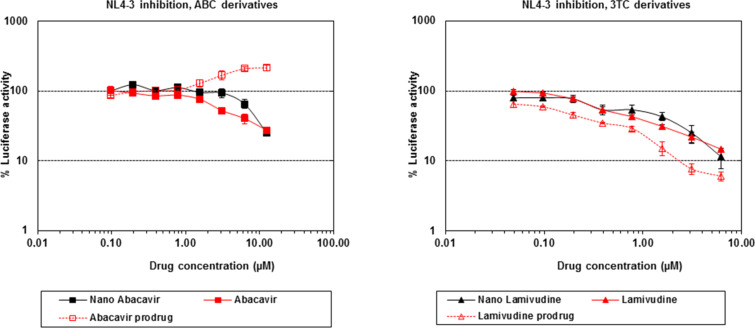
Cellular experiments: The two graphs show the percentage of luciferase activity decrease in the presence of increasing amounts of GNPs. ABC-GNPs (left) show an antiviral activity with an IC_50_ of 8 µM. 3TC–GNPs (right) show an antiviral activity with an IC_50_ of 1 µM.

TZM-bl cells (derived HeLa-cell immortalized cell line that expresses high levels of CD4 and co-receptors CXCR4 and CCR5) were incubated for 30 min with different amounts of drug-GNPs (expressed as drug concentration, from 0.1 to 10 μM), followed by the addition of NL4-3 HIV virus encoding for luciferase used as reporter gene. The free drugs and prodrug candidates were also tested in the same experiment. The viral replication was followed by the luciferase activity setting 100% of viral replication (luciferase activity) for untreated TZM-bl cells. Figure 3 shows the decrease of viral replication (correlated with the percentage of luciferase activity) of the abacavir and lamivudine-GNPs. Free abacavir and the corresponding ABC-GNPs showed similar IC_50_ values of 5 μM and 8 μM, respectively (Figure 3 left and Table 1). Surprisingly, the abacavir derivative seems to induce viral replication. With the presented data we are not able to explain this result, but it may be due to the amphiphilic properties of the drug derivative. Notwithstanding, the inactive abacavir-derivative showed antiviral activity when coupled on GNPs; a similar effect was previously observed for an inactive derivative of TAK-779 [15]. Free lamivudine and the corresponding GNPs showed IC_50_ values of 0.35 μM and 1 μM, respectively (Figure 3 right and Table 1), while the lamivudine derivative showed an IC_50_ value of 0.2 μM. The antiviral activity of the free drugs and the drugs-GNPs were in the same order of magnitude, while the control glucose-GNPs were not able to exhibit any antiviral activity at the tested concentrations (data not shown). In spite of the fact that no improvement of viral replication inhibition was obtained with respect to the free drug (probably due to the low loading of the drugs on the GNPs) these data indicate that the antiviral activity after conjugation is maintained and that gold glyconanoparticles can be considered as a promising drug delivery system.

**Table 1:** Antiviral activity of tested molecules calculated as IC_50_ from the cellular experiments.

Molecule tested	IC_50_

abacavir	5 µM
abacavir derivative	–^a^
abacavir-GNP	8 µM
lamivudine	0.35 µM
lamivudine derivative	0.2 µM
lamivudine-GNP	1 µM

^a^The abacavir derivative showed the ability to induce viral replication.

After 30 min of pre-incubation with TZM-bl cells, the drug-loaded glyconanoparticles showed an NRTi activity as the free drugs at similar concentration. This activity suggests that the drug is delivered from the GNPs into the TZM-bl cells and has been triphosphorylated to active metabolites that can compete with the natural substrate of RT avoiding the RNA retrotranscription, e.g., the viral replication. Abacavir and lamivudine (being NRTi) inhibit the HIV reverse transcriptase enzyme competitively and act as a chain terminator in DNA synthesis. The lack of a 3'-OH group in the nucleoside analogue (NRTi) inhibits the formation of the 5' to 3' phosphodiester linkage (essential for the elongation of the DNA chain) terminating the growth of viral DNA [3].

## Conclusion

The preparation and characterization of ~3 nm glucose-coated gold nanoparticles loaded with anti-HIV abacavir and lamivudine ester prodrug candidates is described. The effects of multimerization of the HIV drug derivatives on biocompatible and water-dispersible glyconanomaterials have been tested. The drugs were released from the glyconanoparticles in acidic conditions and were able to inhibit viral replication in cellular assays with IC_50_ values (in terms of drug concentration) similar to the free drugs (less than 10 µM). These data support the strategy of developing a drug delivery system based on the coupling of ester derivatives onto gold glyconanoparticles and open the way to re-design more complex GNPs with improved activity carrying different antiviral inhibitors at the same time. In addition, other types of molecules able to block different steps of the viral replication can be introduced on the GNPs surface as previously shown with the microbicide candidates sulfate and *manno*-GNPs [19–20]. The combination of the gold glyconanoparticle properties with the advantage of multiple presentations of drugs, opens-up the possibility for generating multivalent nano delivery systems against HIV, combining on the same nanoparticle scaffold different antiviral inhibitors. Further experiments need to be performed to investigate the molecular mechanisms of the described antiviral activity. A cellular tracking of the GNPs could give a molecular explanation of their behavior in the intracellular milieu. The described proof-of-principle aims to a further exploration of gold glyconanoparticles as a new multifunctional tool in the world of drug-delivery system against HIV.

## Experimental

**General methods:** All chemicals were purchased as reagent grade from Sigma-Aldrich, except chloroauric acid (Strem Chemicals), and were used without further purification. NMR analyses were performed with a Bruker DRX 500 MHz spectrometer with a broad band inverse (BBI) probe at 25 °C. Chemical shifts (δ) are given in ppm relative to the residual signal of the solvent used. Coupling constants (*J*) are reported in Hz. Splitting patterns are described by using the following abbreviations: br, broad; s, singlet; d, doublet; t, triplet; q, quartet; m, multiplet. For transmission electron microscopy (TEM) examinations, a single drop (10 µL) of an aqueous solution (ca. 0.1 mg/mL in milli-Q water) of drugs-GNPs was placed onto an ultrathin carbon film (<3 nm thickness) supported by a lacey carbon film on a 400 mesh copper grid (Ted Pella). The solution on the grid was left to dry in air for 14 hours at room temperature. TEM analysis was carried out in a JEOL JEM-2100F-UHR, operated at 200 kV. UV–vis spectra were carried out with a Beckman Coulter DU 800 spectrometer. The mass spectrometry detection was carried out in positive ion mode with electrospray ionization. The capillary and the cove voltages were set to 100 and 30 V, respectively. The desolvation gas was set to 600 L/h at 120 °C. The cone gas was set to 50 L/h and the ion source temperature at 120 °C. The instrument was operated in W mode with a resolution higher than 10.000. Data were obtained in centroid mode from *m*/*z* 50 to 1000 using a acquisition rate of 1 s/scan. The extracted-ion chromatograms for each compound were obtained with a mass tolerance window of ±0.1 Da (*m*/*z* 230.06 for 3TC, *m*/*z* 287.16 for ABC, 244.09 for cytidine, *m*/*z* 205.1 for tryptophan). An Acquity UPLC coupled to LCT Premier XE mass spectrometer (Waters, Mildford, MA) was employed for the drug quantification. The chromatographic separations were performed on a 100 × 2.1 mm Acquity BEH 1.7 µm C18 column (Waters, Mildford, MA). The gradient elution buffers were A (water and 0.1% formic acid) and B (methanol). The column temperature was set to 35 °C and eluted with a linear gradient consisted of 95% A over 0.5 min, 95–5% over 0.5–7 min, 5% over 7–8 min, returned to 95% for 0.5 min and kept for a further 1.5 min before next injection. Total run was 10 min, volume injection 5 µL and the flow rate 300 µL/mL.

**Synthesis and characterization of thiol-ending prodrugs and GNPs:** The preparation and characterization of the abacavir and lamivudine prodrug candidates and the corresponding GNPs is described in the Supporting Information File 1.

**LC–MS analysis**: GNPs and calibration curve samples were spiked with 10 µL of the appropriate internal standard solution before the LC–MS analysis (tryptophan and cytidine at 1 µM were used for quantification of 3TC and ABC, respectively). Calibration curves were designed over the range of 1–200 nM in triplicate. All the standard solutions were above the lower limit of quantification and within a linear range of quantification (R^2^ > 0.998). Peak ratios of the drug and the internal standard were calculated and the calibration curves adjusted by fitting these ratios to the concentrations by a linear regression method.

**Cellular viral inhibition assay:** The ability of lamivudine and abacavir-GNPs to block HIV-1 infection was tested using a luciferase reporter cell line (TZM-bl) as described in [36]. TZM-bl is a Hela cell line that stably expresses CD4, CCR5 and CXCR4 (viral receptor and co-receptors). These cells also contain separate integrated copies of the luciferase and β-galactosidase genes under the control of the HIV-1 promoter [37–40]. Drugs, ester derivatives and GNPs were incubated with HIV-1 virus (NL4-3 strain) in triplicate for 30 min at 37 °C. The virus–drug mixture was added (1:1 by volume) to 10,000 TZM-bl cells per well. The plate was then placed into a humidified chamber within a CO_2_ incubator at 37 °C. The luciferase activity was measured from cell lysates when the levels were sufficiently over the background to give reliable measurements (at least 10 fold) using Luciferase Assay System (Promega) and following the manufacturer’s recommendations. A virus equivalent to 4 ng of p24 capsid protein (quantified by an antigen-capture assay; Innogenetics, Belgium) of the NL4-3 strain of HIV-1 was chosen as the lowest level of viral input sufficient to give a clear luciferase signal within the linear range at day 3 post-infection. Infectivity was measured in triplicate and reported as the percentage of luciferase activity compared to the luciferase activity corresponding to the wells with virus and no drug. The concentration of drug required to inhibit 50% of the viral infectivity (IC_50_) was determined.

## Supporting Information

File 1 Synthesis and characterization of thiol-ending prodrugs and GNPs; HPLC–MS chromatograms, mass spectra and drugs calibration curves; calculation of drug-loading on GNPs.

## References

[1] Gallo R C, Montagnier L (2003). N Engl J Med.

[2] (2014). Data and statistics from WHO webpage.

[3] De Clercq E (2010). Curr Opin Pharmacol.

[4] Munier C M L, Andersen C R, Kelleher A D (2011). Drugs.

[5] Walker B D, Burton D R (2008). Science.

[6] Balzarini J, Van Damme L (2007). Lancet.

[7] Abdool Karim Q, Abdool Karim S S, Frohlich J A, Grobler A C, Baxter C, Mansoor L E, Kharsany A B M, Sibeko S, Mlisana K P, Omar Z (2010). Science.

[8] Allen T M, Cullis P R (2004). Science.

[9] Pelgrift R Y, Friedman A J (2013). Adv Drug Delivery Rev.

[10] Date A A, Destache C J (2013). Biomaterials.

[11] Lisziewicz J, Tőke E R (2013). Nanomed Nanotechnol Biol Med.

[12] Boisselier E, Astruc D (2009). Chem Soc Rev.

[13] Dykman L, Khlebtsov N (2012). Chem Soc Rev.

[14] Duncan B, Kim C, Rotello V M (2010). J Controlled Release.

[15] Bowman M-C, Ballard T E, Ackerson C J, Feldheim D L, Margolis D M, Melander C (2008). J Am Chem Soc.

[16] Elechiguerra J L, Burt J L, Morones J R, Camacho-Bragado A, Gao X, Lara H H, Yacaman M J (2005). J NanoBiotechnology.

[17] Shiang Y-C, Ou C-M, Chen S-J, Ou T-Y, Lin H-J, Huang C-C, Chang H-T (2013). Nanoscale.

[18] Di Gianvincenzo P, Chiodo F, Marradi M, Penadés S (2012). Methods Enzymol.

[19] Martínez-Ávila O, Bedoya L M, Marradi M, Clavel C, Alcamí J, Penadés S (2009). ChemBioChem.

[20] Di Gianvincenzo P, Marradi M, Martínez-Ávila O, Bedoya L M, Alcamí J, Penadés S (2010). Bioorg Med Chem Lett.

[21] Marradi M, Chiodo F, García I, Penadés S (2013). Chem Soc Rev.

[22] Arnáiz B, Martínez-Ávila O, Falcon-Perez J M, Penadés S (2012). Bioconjugate Chem.

[23] de la Fuente J M, Alcántara D, Penadés S (2007). IEEE Trans Nanobiosci.

[24] Irure A, Marradi M, Arnáiz B, Genicio N, Padro D, Penadés S (2013). Biomater Sci.

[25] Murray R A, Qiu Y, Chiodo F, Marradi M, Penadés S, Moya S E (2014). Small.

[26] Moros M, Hernáez B, Garet E, Dias J T, Sáez B, Grazú V, González-Fernández A, Alonso C, de la Fuente J M (2012). ACS Nano.

[27] Safari D, Marradi M, Chiodo F, Dekker H A T, Shan Y, Adamo R, Oscarson S, Rijkers G T, Lahmann M, Kamerling J P (2012). Nanomedicine.

[28] Chiodo F, Marradi M, Tefsen B, Snippe H, van Die I, Penadés S (2013). PLoS One.

[29] Bibby D C, Charman W N, Charman S A, Iskander M N, Porter C J H (1996). Int J Pharm.

[30] Tichý T, Andrei G, Dračínský M, Holý A, Balzarini J, Snoeck R, Krečmerová M (2011). Bioorg Med Chem.

[31] Tahir M N, Théato P, Müller W E G, Schröder H C, Janshoff A, Zhang J, Huth J, Tremel W (2004). Chem Commun.

[32] Barrientos A G, de la Fuente J M, Rojas T C, Fernández A, Penadés S (2003). Chem–Eur J.

[33] Marradi M, Martín-Lomas M, Penadés S (2010). Adv Carbohydr Chem Biochem.

[34] Hostetler M J, Templeton A C, Murray R W (1999). Langmuir.

[35] Martínez-Ávila O, Hijazi K, Marradi M, Clavel C, Campion C, Kelly C, Penadés S (2009). Chem–Eur J.

[36] Marradi M, Di Gianvincenzo P, Enríquez-Navas P M, Martínez-Ávila O M, Chiodo F, Yuste E, Angulo J, Penadés S (2011). J Mol Biol.

[37] Takeuchi Y, McClure M O, Pizzato M (2008). J Virol.

[38] Wei X, Decker J M, Liu H, Zhang Z, Arani R B, Kilby J M, Saag M S, Wu X, Shaw G M, Kappes J C (2002). Antimicrob Agents Chemother.

[39] Derdeyn C A, Decker J M, Sfakianos J N, Wu X, O'Brien W A, Ratner L, Kappes J C, Shaw G M, Hunter E (2000). J Virol.

[40] Platt E J, Wehrly K, Kuhmann S E, Chesebro B, Kabat D (1998). J Virol.

