# Supplementary material for: Glycosystems in nanotechnology: Gold glyconanoparticles as carrier for anti-HIV prodrugs
Source: Beilstein J Org Chem. 2014 Jun 12;10:1339–46. doi: 10.3762/bjoc.10.136 (PMC4077455; doi:10.3762/bjoc.10.136)
Supplement: File 1 — Synthesis and characterization of thiol-ending prodrugs and GNPs; HPLC–MS chromatograms, mass spectra and drugs calibration curves; calculation of drug-loading on GNPs. [file Beilstein_J_Org_Chem-10-1339-s001.pdf]

Supporting Information

for

Glycosystems in nanotechnology: Gold glyconanoparticles as carrier for anti-HIV prodrugs

Fabrizio Chiodo*[1,§], Marco Marradi[1,2], Javier Calvo[3], Eloisa Yuste[4,5] and Soledad Penadés*[1,2]

Address: [1]Laboratory of GlycoNanotechnology, Biofunctional Nanomaterials Unit, CIC biomaGUNE, Paseo Miramón 182, 20009, San Sebastián, Spain, [2]Networking Research Center on Bioengineering, Biomaterials and Nanomedicine (CIBER-BBN), Paseo Miramón 182, 20009, San Sebastián, Spain, [3]Technological Platform of Mass Spectrometry, CIC biomaGUNE, Paseo Miramón 182, 20009, San Sebastián, Spain, [4]AIDS Research Unit, Institut d'Investigacions Biomediques August Pi i Sunyer, Barcelona, Spain and [5]HIVACAT, Barcelona, Spain,

[§]Present address: Department of Parasitology, Leiden University Medical Center, 2333 ZA, Leiden, Netherlands.

Email: Fabrizio Chiodo - chiodo.fabrizio@gmail.com; Soledad Penadés - spenades@cicbiomagune.es

*Corresponding author

Synthesis and characterization of thiol-ending prodrugs and GNPs; HPLC–MS chromatograms, mass spectra and drugs calibration curves; calculation of drug-loading on GNPs.

Synthesis and characterization of thiol-ending prodrugs and GNPs

Abacavir (ABC) derivative synthesis and abacavir-GNPs preparation

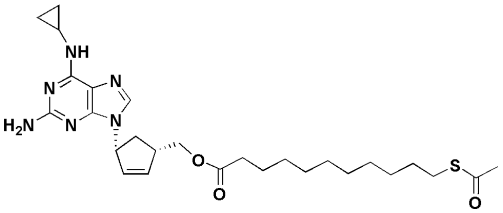

Protected abacavir conjugate: Abacavir (63 mg, 0.2 mmol) was dissolved in dry DMF (3 mL) followed by the addition of 11-(acetylthio)undecanoic acid (89 mg, 0.3 mmol) and DMAP (54 mg, 0.4 mmol). The solution was cooled to 0 ºC with an ice bath and EDC (84 mg, 0.4 mmol) was added. After 5 h the reaction was diluted with AcOEt and washed with water (4 × 5 mL). The organic phase was dried over Na_2SO_4 and purified by column chromatography on silica gel (gradient $MeOH/CH_2Cl_2$ 1/20 to $MeOH/CH_2Cl_2$ 1/9) to

obtain the protected abacavir derivative as a clear oil (100 mg, 0.12 mmol, 85%).^{1}H NMR (500 MHz, MeOD) δ = 7.68 (s, 1H), 6.15-6.12 (m, 1H), 5.97-5.94 (m, 1H), 5.52-5.49 (m, 1H), 4.19-4.11 (m, 2H), 3.20-3.13 (m, 1H), 2.95-2.87 (m, 1H), 2.86-2.79 (m, 1H), 2.29 (s, 3H, SAc), 1.68-1.63 (m, 1H), 1.56-1.49 (m, 4H), 1.35-1.20 (m, 12H), 0.86-0.82 (m, 2H), 0.62-0.59 (m, 2H).

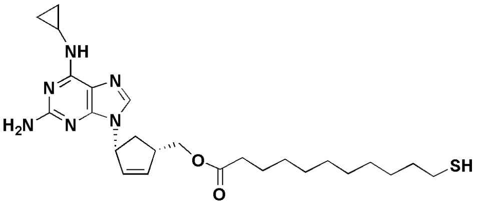

Abacavir derivative (ester prodrug): S-acetyl protected abacavir derivative (65 mg, 0.12 mmol) was dissolved in MeOH (3 mL) and stirred in presence of hydrazine acetate (35 mg, 0.3 mmol). After 24 h the reaction was diluted with AcOEt, neutralized with HCl (0.1 M) and washed with water (5 × 8 mL). The organic phase was dried over Na_2SO_4 and the residue was purified by size-exclusion chromatography Sephadex LH-20 ($MeOH/H_2O$ 9/1) to give the unprotected abacavir derivative (34 mg, 56%). ^{1}H NMR (500 MHz, MeOD) δ = 7.69 (s, 1H), 6.17-6.13 (m, 1H), 5.99-5.95 (m, 1H), 5.53-5.50 (m, 1H), 4.20-4.11 (m, 2H), 3.20-3.12 (m, 1H), 2.96-2.87 (m, 1H), 2.83-2.79 (m, 1H), 2.65 (t, J = 7.2, disulfide ~10%), 2.47 (t, J=7.1, 2H, thiol ~90%), 2.31-2.27 (m, 2H), 1.69-1.64 (m, 1H), 1.59-1.53 (m, 4H), 1.40-1.19 (m, 12H), 0.92-0.80 (m, 2H), 0.65-0.57 (m, 2H). High resolution mass spectrometry, theoretical [M+H] m/z: 487.2856, experimental 487.2854, deviation < 1 ppm.

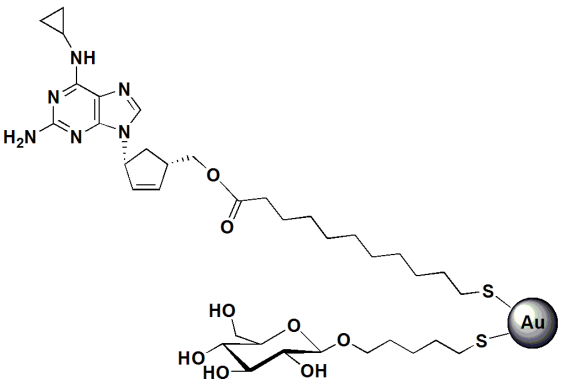

Abacavir GNPs: 1 mg of glucose-GNPs were dissolved in a mixture of H_2O/MeOH (140 µL). 0.046 mg of Abacavir ester prodrug (0.1 equiv with respect to the glucose-GNPs ligands, 90% thiol, 10% disulfide checked by NMR) in MeOH solution were added to the glucose-GNPs. After 22 h of shaking at room temperature the GNPs where precipitated with EtOH and washed 5 times (5 × 1 mL EtOH). The final dark solid was then dissolved in 450 µL of H_2O + 50 µL of DMSO. TEM (average gold diameter): 2.9 ± 0.9 (Figure S1-A); UV–vis (H_2O/DMSO, 0.1 mg/mL): absorbance starts at 800 nm increasing exponentially till 200 nm (Ab s~1.5). The typical surface plasmon band at 520 nm coming from the nanometric gold was not observed cause of the small dimensions of these GNPs.

Lamivudine (3TC) derivative synthesis and lamivudine-GNPs preparation

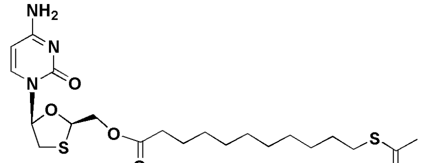

Protected lamivudine derivative: Lamivudine (12 mg, 0.05 mmol) was dissolved in dry DMF (3 mL) followed by the addition of 11-(acetylthio)undecanoic acid (20 mg, 0.07 mmol) and DMAP (12.5 mg, 0.1 mmol). The solution was cooled to 0 ºC with an ice bath and EDC (19.5 mg, 0.1 mmol) was added. After 3 h the reaction was diluted with AcOEt and washed with water (4 × 5 mL). The organic phase was dried over Na_2SO_4 and purified by column chromatography on silica gel (gradient $MeOH/CH_2Cl_2$ 1/40 to $MeOH/CH_2Cl_2$ 1/10) to obtain the protected lamivudine derivative as a clear oil (8 mg, 70%). ^{1}H NMR (500 MHz, MeOD) δ = 7.88 (d, *J*=7.5, 1H), 6.29 (t, *J*=4.9, 1H), 5.90 (d, *J*=7.5, 1H), 5.44-5.42 (m, 1H), 4.61-4.58 (m, 1H), 4.49 (AB system, 2H), 3.54 (dd, *J*=12.0, 5.4, 1H), 3.15 (dd, *J*=12.0, 4.6, 1H), 2.85 (t, J=7.3, 2H), 2.39 (t, *J*=7.3, 2H), 2.30 (s, 3H, SAc), 1.67-1.61 (m, 2H), 1.58-1.52 (m, 2H), 1.38-1.26 (m, 12H).

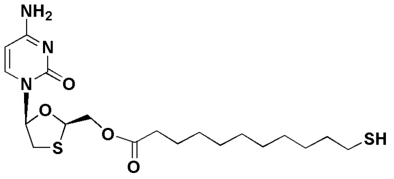

Lamivudine derivative (ester prodrug): S-acetyl protected lamivudine derivative (4 mg, 0.007 mmol) was dissolved in MeOH (2 mL) and stirred in presence of hydrazine acetate (2 mg, 0.02 mmol). After 3 h the reaction was diluted with AcOEt, neutralized with HCl (0.1 M) and washed with water (4 × 4 mL). The organic phase was dried over Na_2SO_4 and the residue was purified by size-exclusion chromatography Sephadex LH-20 ($MeOH/H_2O$ 9/1) to give the unprotected lamivudine derivative (2 mg, 56%). ^1H NMR (500 MHz, MeOD) δ = 7.88 (d, *J*=7.5, 1H), 6.29 (t, *J*=5.0, 1H), 5.91 (d, *J*=7.5, 1H), 5.44-5.42 (m, 1H), 4.49 (AB system, 2H), 3.54 (dd, *J*=12.0, 5.4, 1H), 3.15 (dd, *J*=12.0, 4.6, 1H), 2.48 (t, *J*=7.1, 2H), 2.39 (t, *J*=7.3, 2H), 1.69-1.53 (m, 4H), 1.43-1.25 (m, 12H). High resolution mass spectrometry, theoretical [M+H] m/z: 430.1834, experimental 430.1809, deviation 5.8 ppm.

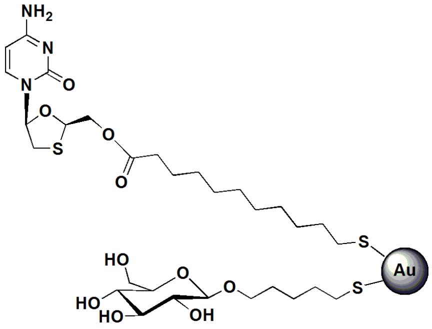

Lamivudine GNPs: 1 mg of glucose-GNPs was dissolved in a mixture H_2O/MeOH (140 µL). 0.040 mg of lamivudine ester prodrug (0.1 equiv with respect to the glucose-GNPs ligands, 90% thiol, 10% disulfide checked by NMR) in MeOH solution were added to the glucose-GNPs. After 22 h of shaking at room temperature the GNPs where precipitated with EtOH and washed 5 times (5 × 1 mL EtOH). The final dark solid was then dissolved in 450 µL of H_2O + 50 µL of DMSO. TEM (average gold diameter): 3.0 ± 1.2 nm (Figure S1-B); UV–vis (H_2O/DMSO, 0.1 mg/mL): absorbance starts at 800 nm increasing exponentially till 200 nm (Abs ~1.5). The typical surface plasmon band at 520 nm coming from the nanometric gold was not observed cause of the small dimensions of these GNPs.

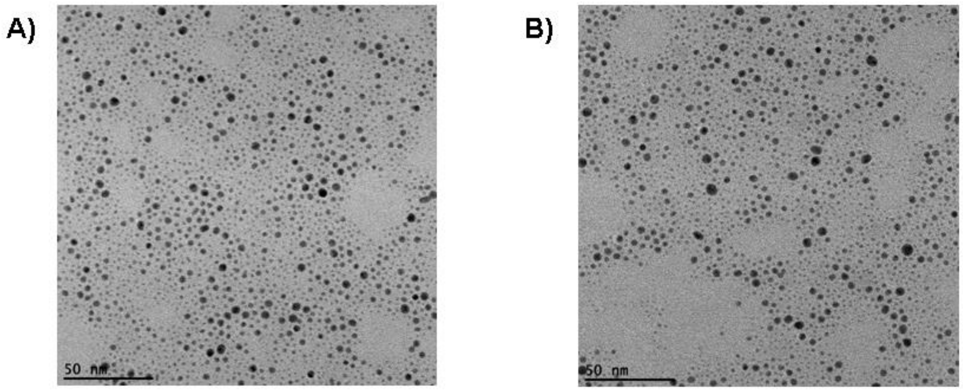

Figure S1: TEM micrographs of the drugs GNPs. **A**) GNPs bearing ABC conjugate, **B**) GNPs bearing 3TC conjugate.

HPLC–MS chromatograms, mass spectra and drugs calibration curves

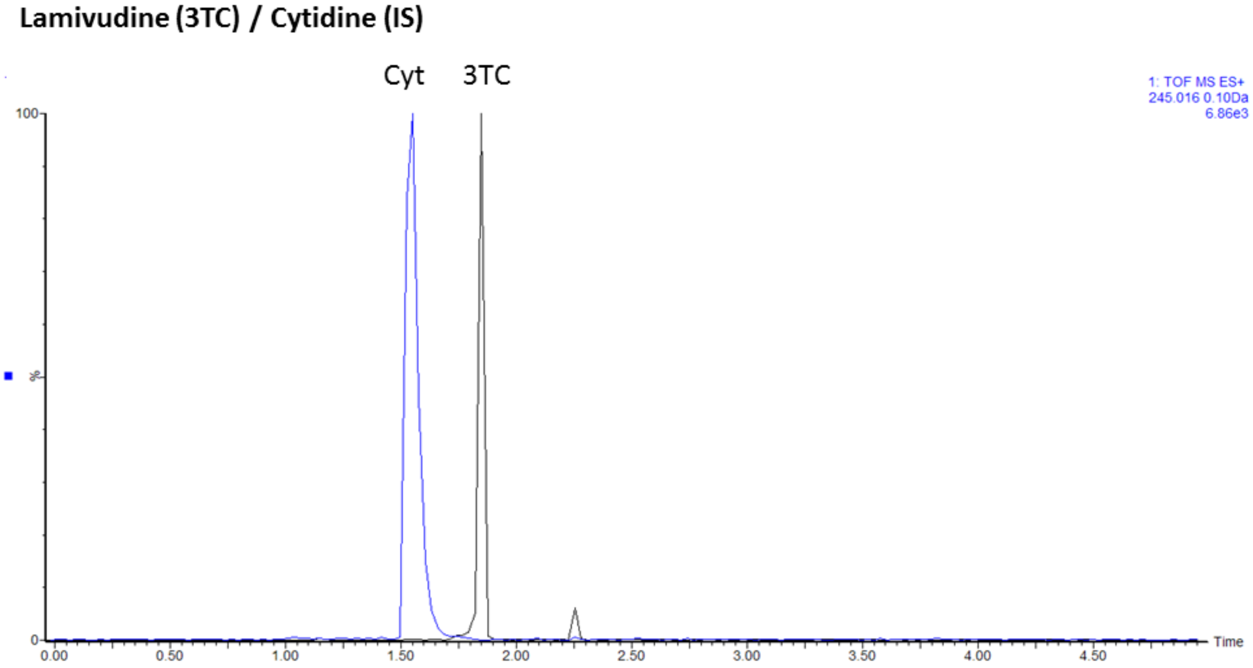

Figure S2: Extracted-ion chromatograms of lamivudine (3TC) and cytidine (internal standard, IS) from 3TC-GNPs treated with HCl (1 M) after 48 h.

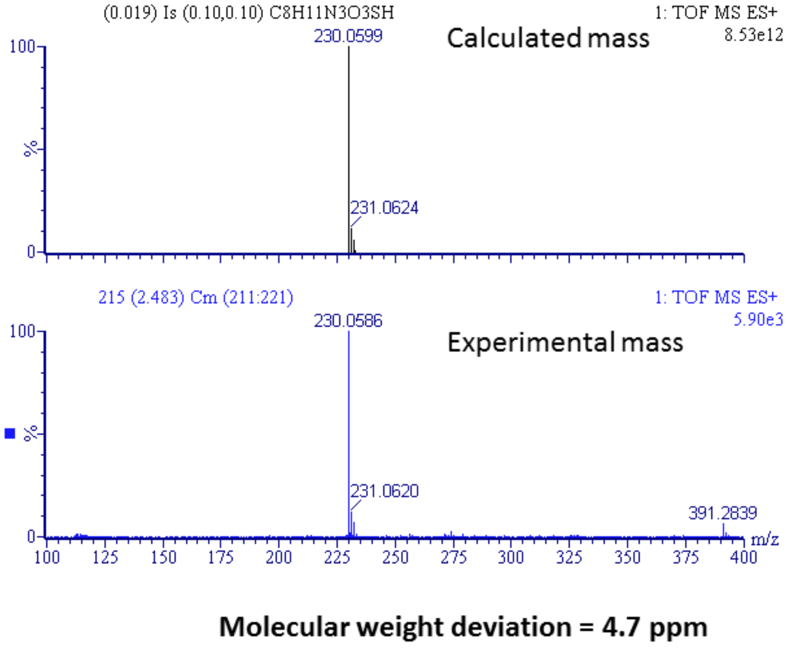

Figure S3: High resolution TOF, mass spectrum from the detected free Lamivudine (3TC) after 1 M HCl treatment of 3TC-GNPs.

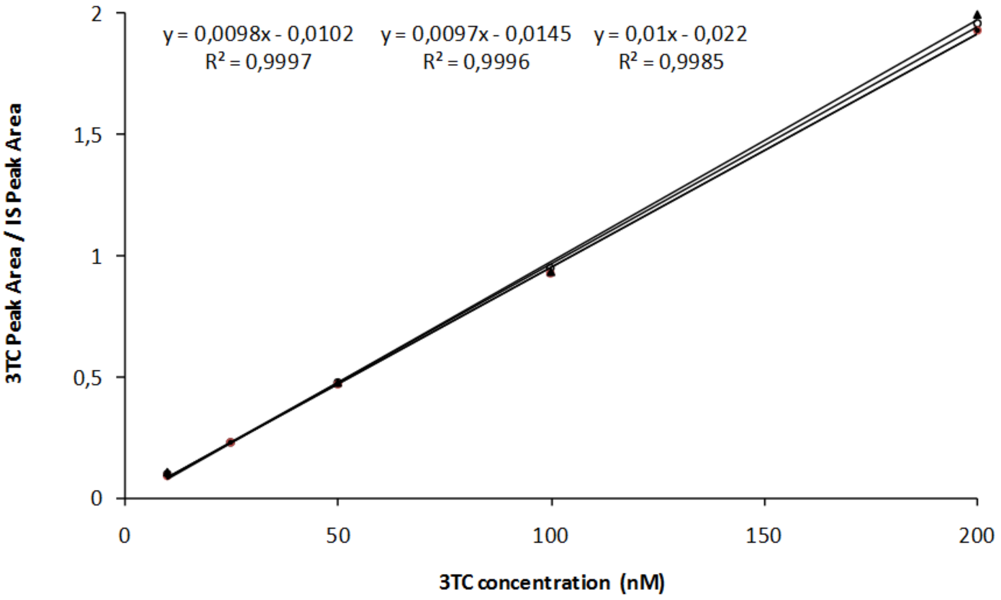

Figure S4: Calibration curve of lamivudine (3TC) after HPLC injection (in triplicate). Peak ratios of the drug and the internal standard (IS) were calculated and the calibration curves adjusted by fitting these ratios to the concentrations by a linear regression method.

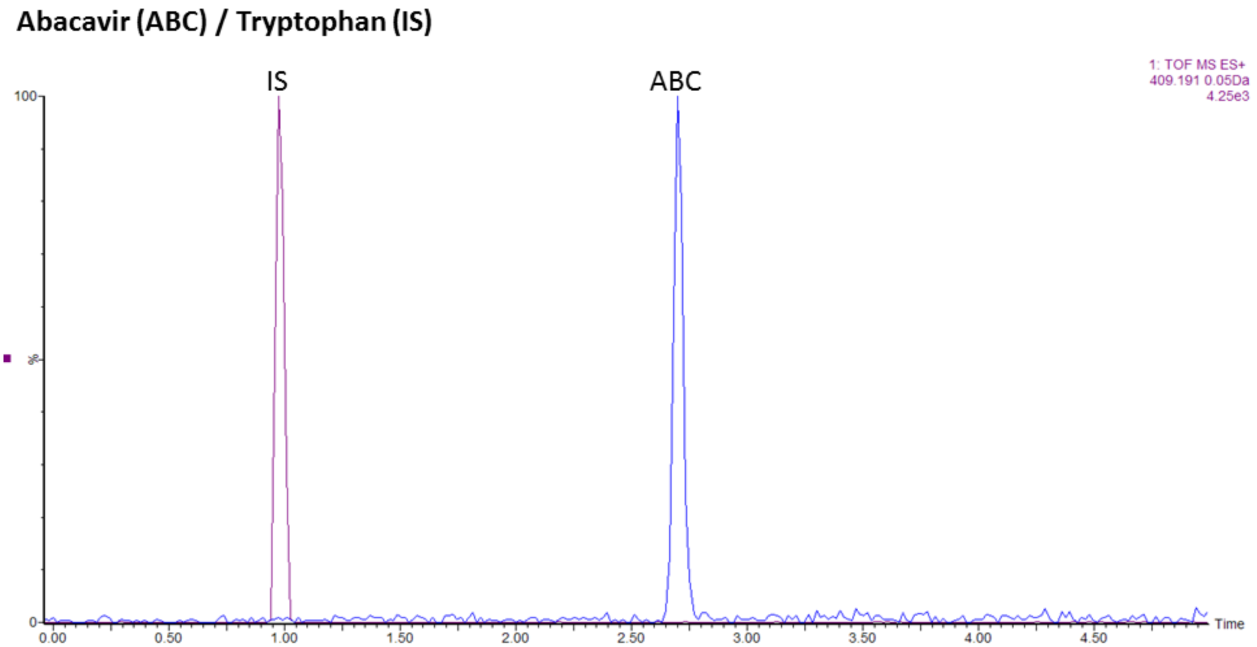

Figure S5: Extracted-ion chromatograms of abacavir (ABC) and tryptophan (internal standard, IS) from ABC-GNPs treated with HCl (1 M) after 48 h.

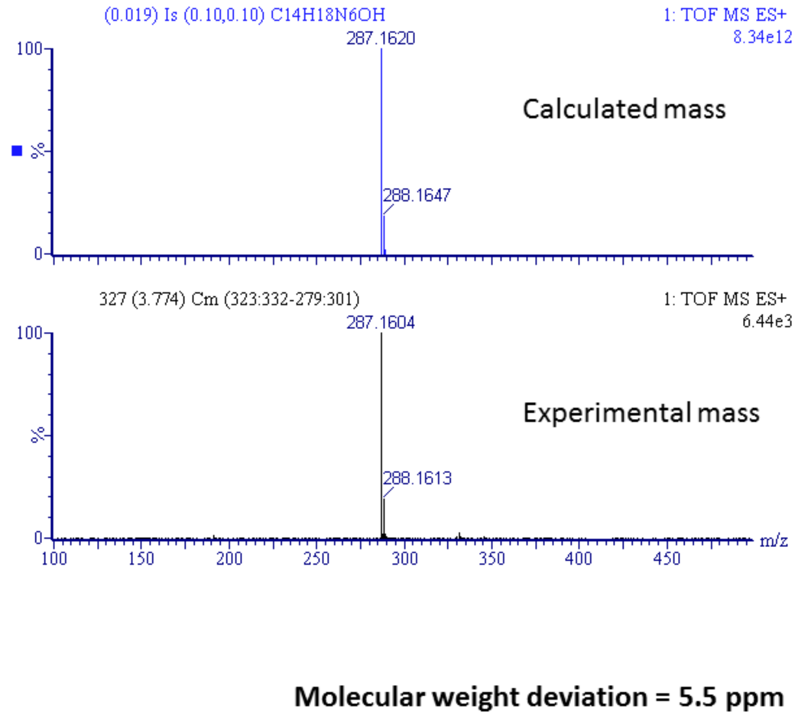

Figure S6: High resolution TOF, mass spectrum from the detected free abacavir (ABC) (1 M) after HCl treatment of ABC-GNPs.

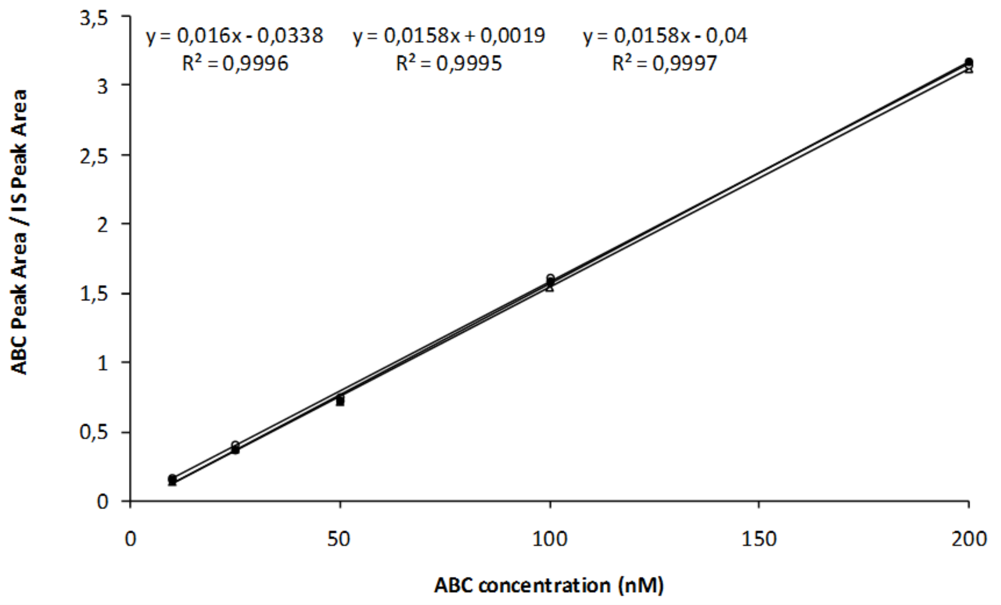

Figure S7: Calibration curve of abacavir after HPLC injection (in triplicate). Peak ratios of the drug and the internal standard (IS) were calculated and the calibration curves adjusted by fitting these ratios to the concentrations by a linear regression method.

Calculation of drug-loading on GNPs.

The average MW of glucose-GNP is 37 KDa with an estimated molecular formula of $Au_{140}(C_{11}H_{21}O_6S)_{35}$ [1]. 1 mg of glucose-GNP = ~0.027 μmol of GNPs = ~0.94 μmol of glucose. We considered that the 0.1 equiv (respect to glucose) of the drug derivatives were linked on the gold surface because we did not observe drug derivatives in the GNPs washings. Then, we assumed that 1 mg of drug-GNPs carries ~0.094 μmol of drugs.

From LC–MS analysis, we measured that in 2 μg/mL of drug-GNPs the drug concentration was ~200–300 nM (after GNPs treatment at pH 0 for several days). In more details, 2 mg of drug-GNPs were treated with 1 mL of HCl (1 M), and then diluted 1000 times; we found 0.2 nmol of drug after 1000 times dilution before LC–MS analysis. We can finally calculate 0.2 μmol of drug in 1 mL of drug-GNPs solution (2 mg/mL). In conclusion we can estimate that 1 mg of drug-GNPs has 0.1 μmol of drug.

Reference

[1] Tahir, M. N.; Théato, P.; Müller, W. E.; Schröder, H. C.; Janshoff, A.; Zhang, J.; Huth, J.; Tremel, W. *Chem. Commun.* **2004,** 2848–2849. doi:10.1039/b410283e
